# Improving RNAi in the fall armyworm, *Spodoptera frugiperda*

**DOI:** 10.3389/finsc.2026.1820222

**Published:** 2026-06-30

**Authors:** Anil Kumar Moola, Subba Reddy Palli

**Affiliations:** Department of Entomology, Martin Gatton College of Agriculture, Food and Environment, University of Kentucky, Lexington, KY, United States

**Keywords:** EGCG, IAP, nanoparticles, PLL, RNAi, *Spodoptera frugiperda*

## Abstract

The rising incidence of resistance to conventional chemical insecticides necessitates the development of novel, sustainable pest management strategies. RNA interference (RNAi) offers a highly specific alternative; however, its practical application in lepidopteran pests is often limited by low efficacy due to rapid dsRNA degradation, inefficient transport of dsRNA to the site of action, and suboptimal target gene selection. While nanoformulated dsRNA has been shown to improve RNAi efficiency, delivery enhancement alone does not consistently result in high mortality. In the current study, we used PLL/EGCG/dsRNA nanoformulations to improve RNAi-based control of *S. frugiperda*. We first validated the effectiveness of nanoparticle-mediated RNAi in *S. frugiperda* by targeting the inhibitor of apoptosis (IAP) gene. Nanoformulated dsRNA targeting IAP resulted in substantial gene silencing, with an 80% reduction in target gene mRNA levels and 63% larval mortality, confirming that improved dsRNA delivery can significantly enhance RNAi efficacy in lepidopterans. We extended our analysis to evaluate an additional 14 candidate genes selected from recent genome-wide RNAi screens that identified superior RNAi targets. Orthologs of these genes in S. frugiperda, including proteasome subunit β-type 4 (BT4) and 60S ribosomal protein L11 (RPL11), were targeted using PLL/EGCG/dsRNA nanoformulations. Although these genes induced moderate larval mortality, their effects were consistently lower than those observed with IAP, indicating that IAP remains a more effective RNAi target among the genes evaluated in *S. frugiperda*.

## Introduction

As global food demand continues to rise, the widespread emergence of resistance to conventional chemical insecticides poses a significant challenge to sustainable agriculture (1). Prolonged reliance on a limited number of insecticide modes of action has led to the development of resistance in economically important insect pests, while simultaneously raising concerns about environmental contamination, adverse effects on non-target organisms, and potential risks to human health (2). Conventional chemical control strategies face similar limitations; consequently, there is a need for alternative pest management approaches that are more selective, environmentally benign, and compatible with integrated pest management (IPM) programs (3).

Though RNA interference (RNAi) efficacy varies greatly across insect orders, RNAi-based solutions have emerged as a promising, species-specific strategy for insect pest management. Coleopteran insects typically exhibit strong systemic RNAi responses, whereas many lepidopteran species show weak or inconsistent responses to orally delivered dsRNA (4). RNAi research over the past 25 years has emphasized that variability in RNAi efficacy among insect orders is largely driven by differences in dsRNA uptake, degradation, and systemic spread, with lepidopterans among the most responsive (4, 5). RNAi efficiency is often limited by rapid degradation of naked dsRNA in the gut lumen and hemolymph, as well as inefficient uptake by midgut epithelial cells in lepidopteran pests such as *S. frugiperda* (4, 6). Consequently, ingestion of naked dsRNA often results in partial gene knockdown and low mortality, even when targeting essential genes (7). These limitations have been repeatedly emphasized as key obstacles for RNAi-based pest control in Lepidoptera (4).

Given these challenges, there is an urgent need to develop improved dsRNA delivery platforms to enhance RNAi efficacy in insect species with weak systemic RNAi responses (4). Among the proposed solutions, nanoparticle delivery, in particular, has emerged as a promising approach for protecting dsRNA (8) from enzymatic degradation and facilitating its uptake into insect cells. Nanocarriers such as chitosan-based nanoparticles, polymeric and non-polymeric complexes, liposomes, and clay nanosheets can bind dsRNA through electrostatic interactions (9) and protect dsRNA and/or siRNA molecules from enzymatic degradation and promote their translocation across cell membranes, thereby increasing their stability and persistence in the insect gut (3, 10, 11).

Beyond protection, nanoformulations can significantly enhance cellular uptake and intracellular trafficking of dsRNA. Nanoparticles are predominantly taken up via endocytosis and can promote endosomal escape, thereby increasing the availability of dsRNA for processing by the RNAi machinery (12). In *S. frugiperda*, nanoparticle-assisted dsRNA delivery has been shown to result in stronger gene silencing, reduced larval growth, and higher mortality compared with naked dsRNA treatments (12, 13). Although improvements in dsRNA delivery, including nanoformulations, can substantially enhance RNAi efficiency, increasing delivery alone does not always yield consistent or high mortality, particularly when suboptimal target genes are selected. Accumulating evidence indicates that the choice of target gene is a critical determinant of RNAi efficacy and ultimately insect lethality. Consequently, identifying highly potent and reliable RNAi target genes is a key element in developing RNAi-based pest management strategies.

A recent genome-wide RNAi study in the red flour beetle, *Tribolium castaneum*, established a clear method for identifying the best RNAi target genes and pathways for insect control. Using large-scale screening, Buer et al. identified many genes that caused death upon silencing and then narrowed them down to the most effective targets through further testing and clustering analysis (14). The authors further proposed that these superior targets could facilitate the transfer of RNAi-based pest control strategies from a highly RNAi-responsive model insect to other pest species. In the present study, we aimed to identify the target gene by using orthologs of genes identified in genome-wide RNAi screens across various insects, and to enhance RNAi efficacy in *S. frugiperda* through nanoparticle-mediated dsRNA delivery. Selected orthologous genes identified as superior RNAi targets were synthesized as dsRNA and conjugated with nanoparticles to improve mortality. The efficacy of nanoparticle-delivered dsRNA was evaluated by measuring larval mortality in *S. frugiperda*, providing insights into the potential of this integrated RNAi-based pest management approach at the greenhouse level.

## Materials and methods

### Insect rearing

*Spodoptera frugiperda* eggs were purchased from Benzon Research Inc. (Carlisle, PA) and reared in the laboratory on artificial diet at 27 ± 1 °C with a 65 ± 5% humidity, and a photoperiod of 14 h light: 10 h dark cycle. Pupae were sexed, and 10 males and 10 females were kept together in a single cage. After emergence, moths were fed on 10% sugar water.

### Materials

Poly-L-lysine (PLL) 15−30 kDa and the natural polyphenol (−)-epigallocatechin gallate (EGCG) were purchased from Sigma-Aldrich. The PLL stock solution was prepared by dissolving 10 mg/mL of poly-L-lysine (15−30 kDa) in 1 mL of double-distilled water and sonicating in a bath sonicator for 10 minutes. The EGCG stock solution was prepared in double-distilled water at 10 mg/mL concentration.

### dsRNA synthesis and preparation of nanoformulations

#### dsRNA synthesis

Gene-specific primers were designed within a single exon of selected genes. The PCR amplicons were purified using the QIAquick PCR purification kit (QIAGEN). The purified DNA was used as a template for dsRNA synthesis using the Megascript T7 RNA Synthesis Kit (Life Technologies, Carlsbad, CA).

### PLL/EGCG/dsRNA nanoformulations

Cationic polymer, natural polyphenol, and dsRNA nanoparticles were assembled via a two-step self-assembly process, as described previously (8). Initially, 60 μL of dsRNA (50 μg) was mixed with 15 μL of (−)-epigallocatechin gallate (EGCG; 150 μg), and mixed by vortexing and incubated for 30 min at room temperature with gentle agitation. Subsequently, 25 μL of Poly-L-lysine (PLL; 250 μg) was added to the EGCG/dsRNA complex at a 1:3:5 ratio, followed by vortexing and incubate for 30 min and sonication for 5 min.

### Characterization of nanoparticles

#### Dynamic light scattering analysis

The hydrodynamic diameters, zeta potentials, and polydispersity of the binary and tertiary nanoparticles were measured in DI water (1 µg/mL) using a Zetasizer Nano ZS90 (Malvern, England) at 25 °C.

#### Gel retardation assay

The binding of dsRNA to the binary and tertiary nanoparticles was evaluated by 1.0% agarose gel electrophoresis at 120 V for 40 minutes in Tris/borate/EDTA buffer (pH 8.0). The dsRNA bands were visualized under a UV transilluminator at 365 nm after staining with GelRed. Naked dsRNA was used as the control, and a 1 kb Plus DNA ladder was used as a size marker. The synthesized nanoparticles were subsequently used for further assays.

### *In vitro* stability assay of dsRNA and PLL/EGCG nanoparticles in midgut lumen extract

Fresh midgut lumen contents were prepared from FAW. Larvae were chilled on ice for 20 min and dissected under sterile, RNase-free conditions. Briefly, larvae were surface-sterilized with 70% ethanol, and the midgut was carefully isolated in cold RNase-free buffer. Lumen contents were gently extruded into pre-chilled RNase-free microcentrifuge tubes and pooled from multiple larvae to minimize biological variability. The crude lumen extract was clarified by centrifugation at 8,000 × g for 10 min at 4 °C, and the resulting supernatant was used immediately for downstream assays. For the ex vivo dsRNA stability assay, reaction mixtures (20 μL total volume) containing 1 μg (10 μl) of either naked dsRNA or PLL/EGCG/dsRNA nanoparticles were incubated with freshly prepared midgut lumen supernatant (5 μL). Samples were incubated at 25 °C and collected at predetermined time points (0, 15, and 30 min; 1, 2, and 3 h) to evaluate the protection of dsRNA by nanoparticles. To recover dsRNA from PLL/EGCG nanoparticles, sodium dodecyl sulfate (SDS), a negatively charged amphiphilic surfactant, was used to disrupt electrostatic interactions between PLL/EGCG and dsRNA. Briefly, 5 μL of 1% SDS was added to 5 μL of the nanoparticle suspension, and the mixture was incubated at 25 °C for 12 min. Recovered nucleic acids were mixed with RNA loading dye and analyzed by electrophoresis on 1.5% agarose gels at 80–100 V for 30–45 min. Gel images were used to assess dsRNA integrity and protection over time.

### dsRNA bioassays

Corn seedlings were grown under greenhouse conditions and harvested approximately 10 days after germination, at the stage when one fully expanded leaf (~5 cm in length) has developed. Seedlings of uniform size were used in each experiment. A 5 μL aliquot of Triton-X (1:1000 dilution) is applied to both surfaces of the leaf and allowed to dry for 10–15 minutes. Preliminary dose-response experiments were conducted using corn seedlings treated with 1 µg of PLL/EGCG/dsIAP or PLL/EGCG/dsGFP (control) per plant, with one neonate larva released onto each plant. Treatments were applied daily for five days, and treated foliage was provided to larvae maintained in fabric-vented containers. Larval mortality was monitored daily. Although mortality was observed under these conditions, repeated container openings during treatment led to larval escape, thereby affecting assay consistency. To minimize handling-related mortality and improve reproducibility, the experimental design was modified. Corn seedlings were subsequently treated with a single application of 20 µL (10 µg dsRNA) per treatment and allowed to dry for 10–15 minutes. Subsequently, two neonate larvae were released per plant and allowed to feed until the treated foliage was completely consumed. After the plant material was depleted, larvae were individually transferred to cups containing artificial diet and monitored for survival and development until pupation. This modified protocol reduced handling-associated variability and produced more reproducible results. After complete consumption of the treated leaf, the larvae were transferred individually to cups containing artificial diet and monitored for mortality until pupation and adult emergence.

### Real‐time quantitative polymerase chain reaction

Total RNA was isolated from FAW larvae using TRIzol (Molecular Research Center Inc., Cincinnati, OH) and subsequently treated with DNase I (Ambion Inc., Austin, TX). Two micrograms of total RNA were used for first-strand cDNA synthesis with M-MLV Reverse Transcriptase (Invitrogen, USA). The synthesized cDNA was diluted 1:5 in distilled water, and 2 µL was used as the template for RT-qPCR analysis. Each RT-qPCR reaction (10 µl final volume) contained 5 µl of Fast Start SYBR Green Master mix (Roche Diagnostics, Indianapolis), 2 µl of cDNA, and 0.4 µl each of 10 µM forward and reverse gene-specific primers. The amplification parameters consisted of an initial incubation at 95 °C (3 min), followed by 40 cycles of 95 °C (10 sec), 55 °C (20 sec), and 72 °C (30 sec). At least three independent biological replicates were used. Relative mRNA expression levels were calculated using 28S rRNA as the reference gene and the 2-ΔΔCT method.

### Statistical analysis

The statistical significance of the data was analyzed using one-way analysis of variance (ANOVA). Differences among treatments were evaluated using Tukey’s multiple comparison test. P values less than 0.05 were considered statistically significant. All statistical analyses were performed using SPSS (version 12.0) and R Studio.

## Results

### PLL/EGCG/dsRNA nanoformulation

**Figure 1:**
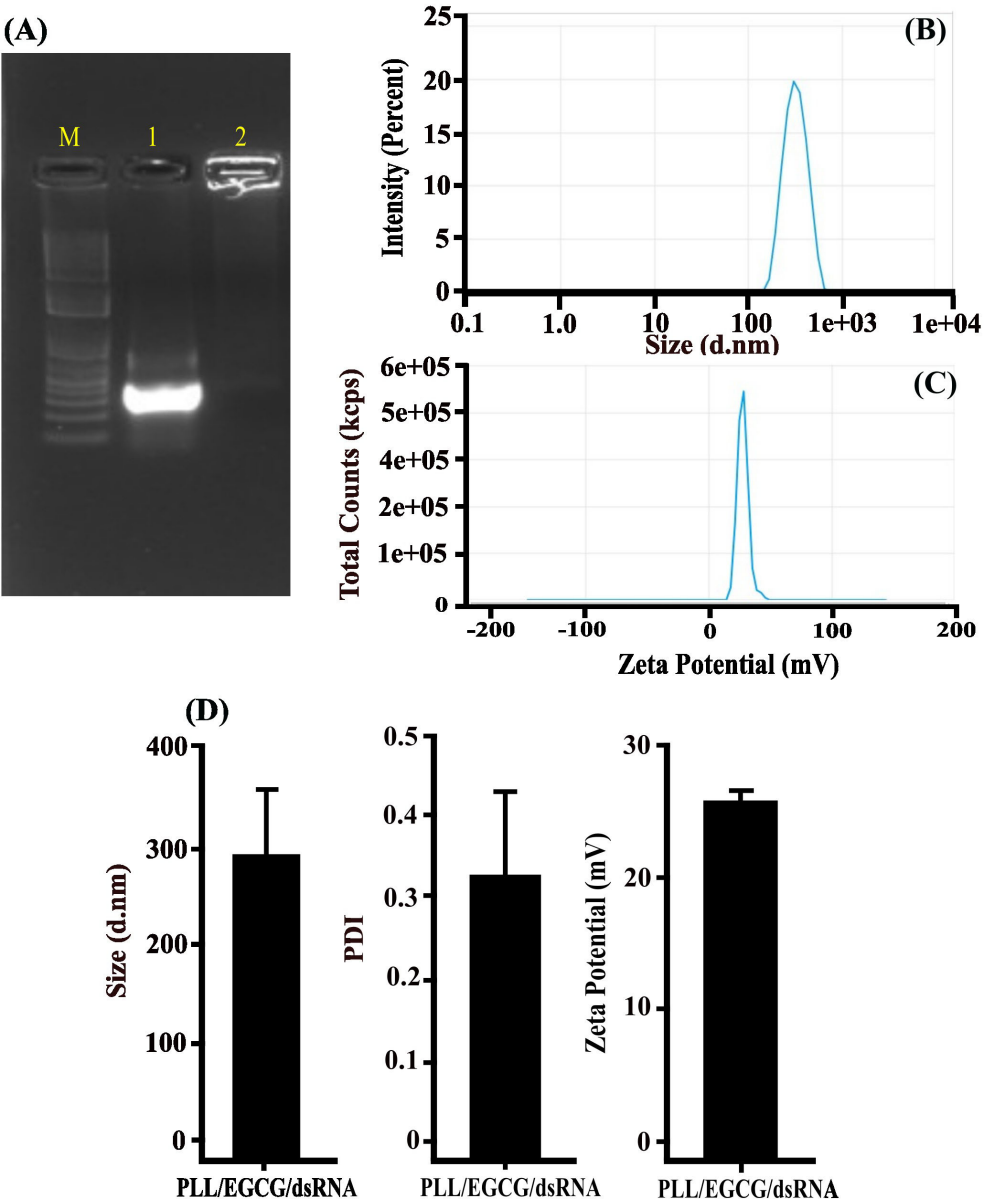
Preparation and characterization of PLL/EGCG/dsRNA nanoparticles. **(A)** The formation of PLL/EGCG/dsRNA complexes was verified by agarose gel electrophoresis. 1 kb plus ladder (Lane 1), naked dsRNA (Lane 2) and PLL/EGCG/dsRNA (Lane 3) complexes were resolved on 1% agarose gels. The gels were stained with GelRed^®^. The gels were photographed under iBrightTM FL1500 image system. **(B–D)** The mean particle diameter (z-average), poly dispersity index (PDI) and zeta potential (surface charge) of PLL/EGCG/dsRNA nanoparticles were determined by DLS analysis (Malvern Instruments, UK). R Studio was used to make graphs.

Building on our previous findings, wherein PLL/EGCG/dsRNA nanoparticles efficiently induced gene knockdown in Sf9 cells (8), the present study evaluated these nanoformulations for dsRNA delivery efficiency and the resulting mortality in *S. frugiperda* larvae. In gel retardation assay, no detectable free dsRNA in the nanoparticles (NPs), in contrast to the naked dsRNA control, suggesting that the dsRNA was completely electrostatically bound to the PLL/EGCG complex (**Figure 1**), possibly due to the presence of polyanions that facilitate the dissociation of nucleic acids (15). The retention of positive surface charge after association with dsRNA also indicates that the complexes were tightly bound, as they do not readily dissociate upon dilution. DLS analysis revealed that the PLL/EGCG/dsRNA complexes exhibited particle sizes of 200–400 nm, a polydispersity index (PDI) of 0.1-0.4, and a zeta potential of 20–30 mV (**Figure 1**; **Supplementary Table 1**).

### Evaluation of stability of PLL/EGCg/dsRNA

**Figure 2:**
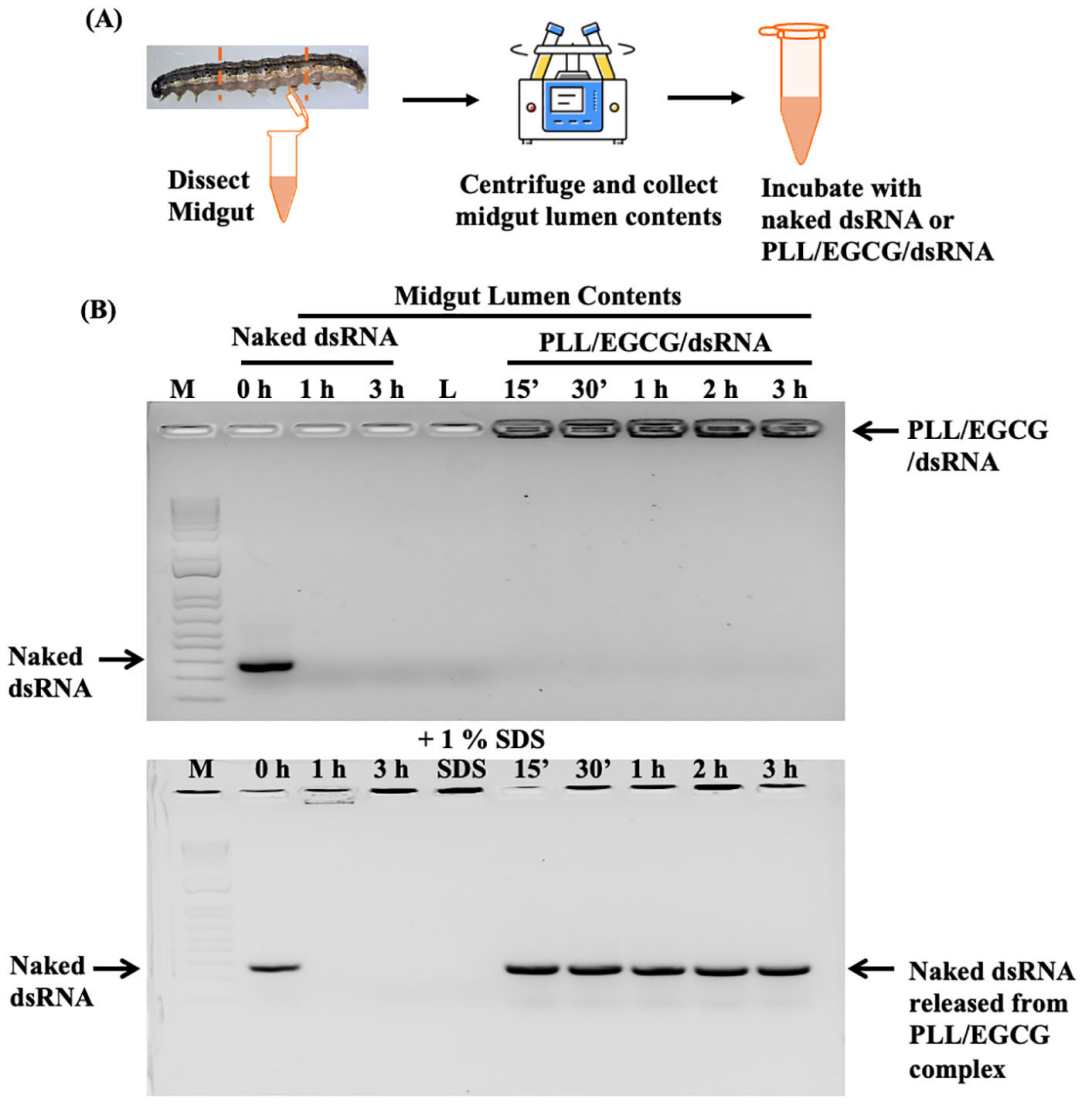
Stability of PLL/EGCG/dsRNA complexes exposed to midgut lumen contents **(A)** Illustration of preparation and evaluation of midgut lumen contents. **(B)** The naked dsRNA and PLL/EGCG/dsRNA exposed to lumen contents for various lengths of time were separated on agarose gels, stained with gel red and photographed. **(C)** The dsRNA released from the naked dsRNA and PLL/EGCG/dsRNA exposed to lumen contents was separated on agarose gels, stained with gel red and photographed. M, marker, 1 kb ladder; L, lumen content.

To evaluate the protection of dsRNA complexed with PLL/EGCG in the midgut lumen of *S. frugiperda*, naked dsRNA or PLL/EGCG/dsRNA nanoparticles were incubated in freshly prepared lumen contents at 27 °C. Naked dsRNA showed signs of degradation within 15 min of incubation (**Figure 2**) and was completely degraded within 1 h. In contrast, dsRNA complexed with PLL/EGCG exhibited no noticeable degradation throughout the 3 h incubation period. These results demonstrate that PLL/EGCG nanoparticles effectively protect dsRNA from degradation in the midgut lumen environment and significantly enhance dsRNA stability compared with naked dsRNA. Recovery of intact dsRNA following SDS treatment at all tested time points further demonstrated that PLL/EGCG nanoparticles effectively protected dsRNA from degradation by the midgut lumen extract for up to 3 h.

### Establishment and validation of a whole-seedling bioassay

**Figure 3:**
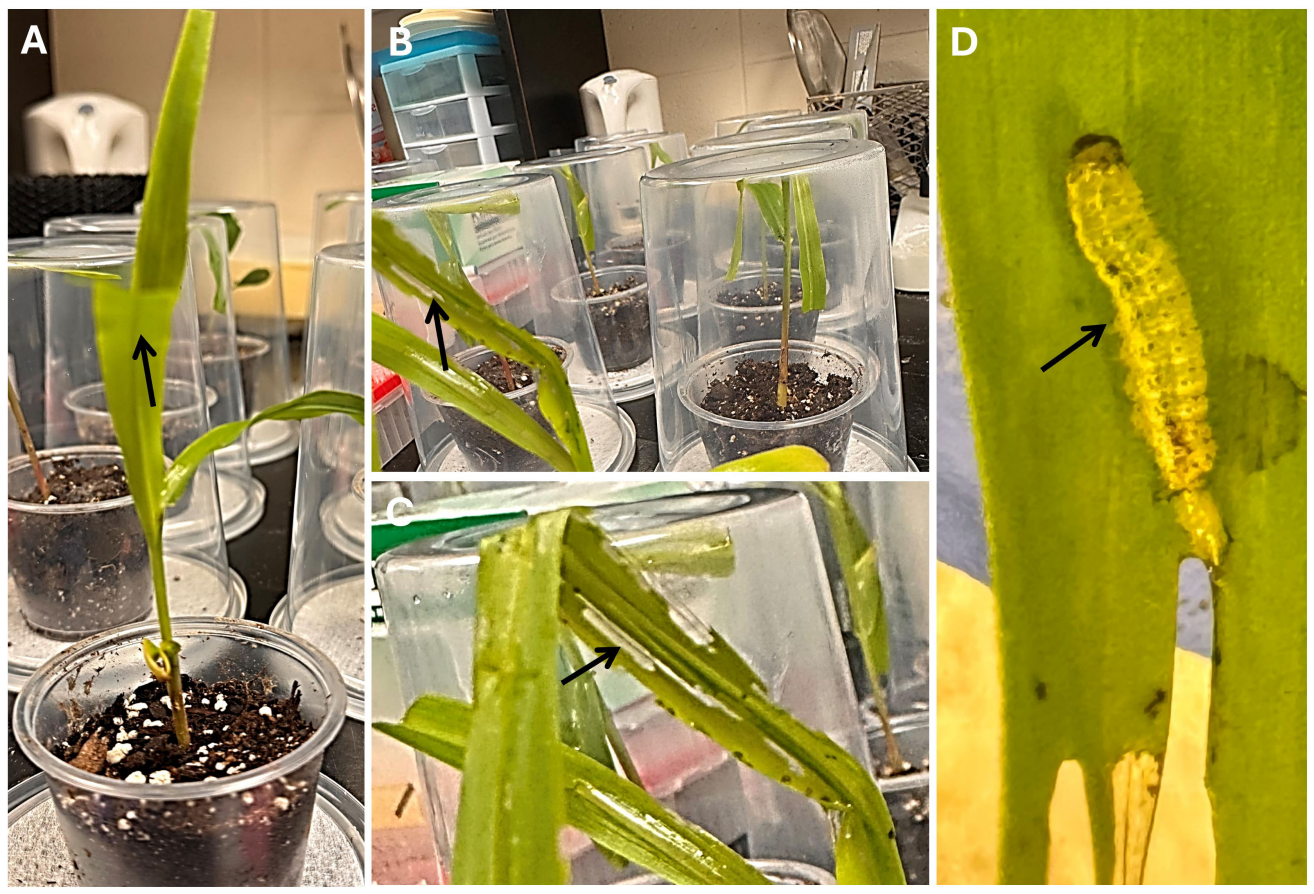
Bioassay of nanoformulated dsRNA. **(A)** Overview of the experimental setup showing corn seedlings in controlled-ventilation growth chambers. The black arrow indicates the target leaf area of application. **(B)** Seedling growth under laboratory conditions. **(C)** Close-up of leaf tissue showing initial feeding marks (indicated by black arrow) post-treatment. **(D)** Detailed view of *Spodoptera* larval feeding behavior; the panel illustrates extensive foliar damage and the presence of fecal frass, confirming active ingestion of the plant material treated with nanoformulated dsRNA.

**Figure 4:**
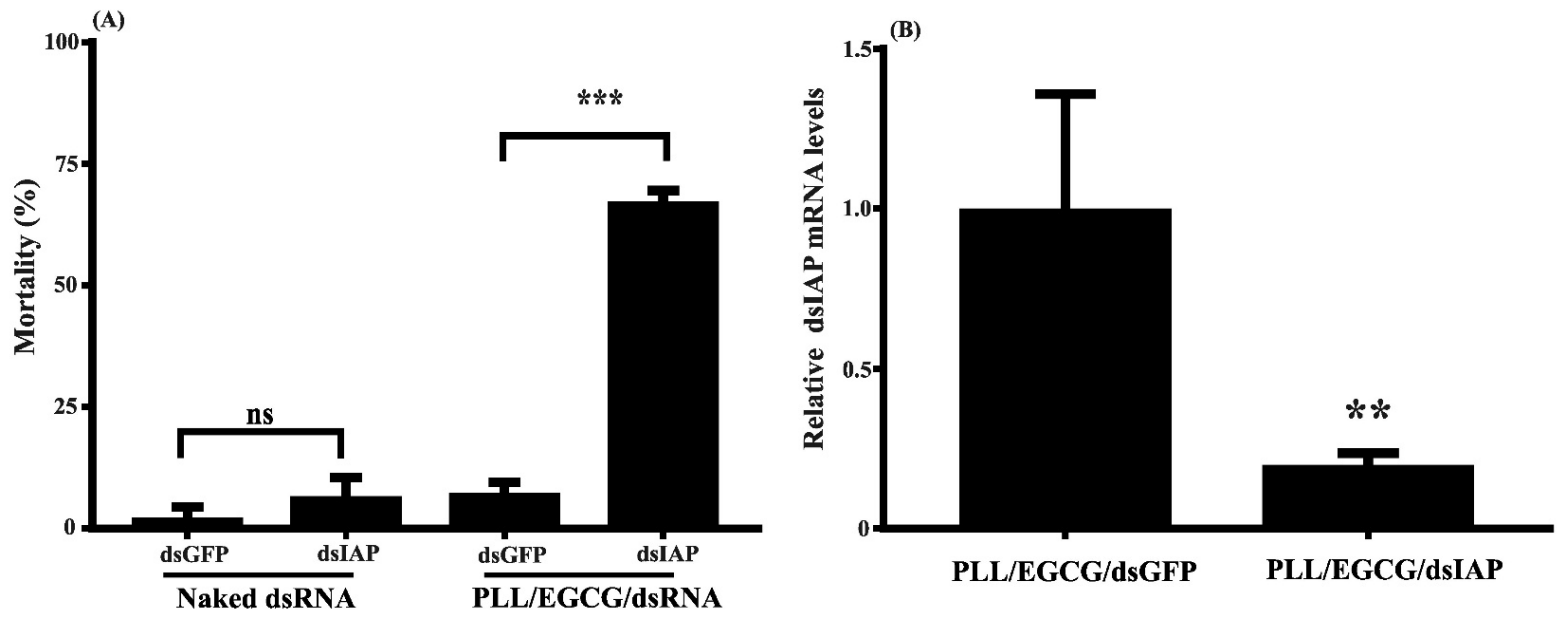
Effect of dsRNA delivery on larval mortality and IAP transcript levels. **(A, B)** Mortality of *S. frugiperda* larvae treated with naked dsRNA (dsGFP or dsIAP) and Mortality of larvae treated with PLL/EGCG nanoformulated dsRNA (dsGFP and dsIAP). For mortality assays, four independent experiments were conducted (n=60). **(B)** Relative IAP mRNA expression in larvae treated with PLL/EGCG/dsGFP and PLL/EGCG/dsIAP. The mRNA levels were quantified by qPCR and normalized to 28S rRNA as the internal reference gene. Relative expression values (2^(-delta Ct) were derived from four independent biological experiments. Bars represent the mean ± SE. Student’s t-test (p < 0.01, **p < 0.001, ***P < 0.001) was used to determine statistical significance. R Studio was used to plot images.

To establish a reliable baseline for RNAi-mediated mortality, the inhibitor of apoptosis (IAP) gene was initially selected as a reference target using nanoparticle-conjugated dsRNA. To evaluate the practical applicability of the delivery system, candidate genes were assessed using a whole-seedling feeding assay. Unlike the leaf-disc method, which primarily measures larval mortality, the whole-seedling approach enabled simultaneous assessment of plant protection and a reduction in feeding damage (**Figure 3**). Following bioassay optimization, the IAP gene was further evaluated as a benchmark RNAi target. Using the optimized nanoformulated delivery system, larval mortality consistently plateaued at approximately 64% (*n* = 60), indicating a strong but incomplete phenotypic response. In contrast, treatment with naked dsRNA did not result in significant mortality. Quantitative PCR analysis revealed a substantial reduction in IAP transcript levels, with approximately 80% knockdown observed in larvae treated with PLL/EGCG/dsIAP compared with control larvae treated with PLL/EGCG/dsGFP (**Figure 4**). These results indicate that the nanoformulated dsRNA was effectively delivered and capable of triggering RNAi-mediated gene silencing in *S. frugiperda*. Together, these findings establish IAP as a robust reference RNAi target and provide a validated benchmark for evaluating additional candidate genes in subsequent screening assays.

### Selection of RNAi target genes

**Figure 5:**
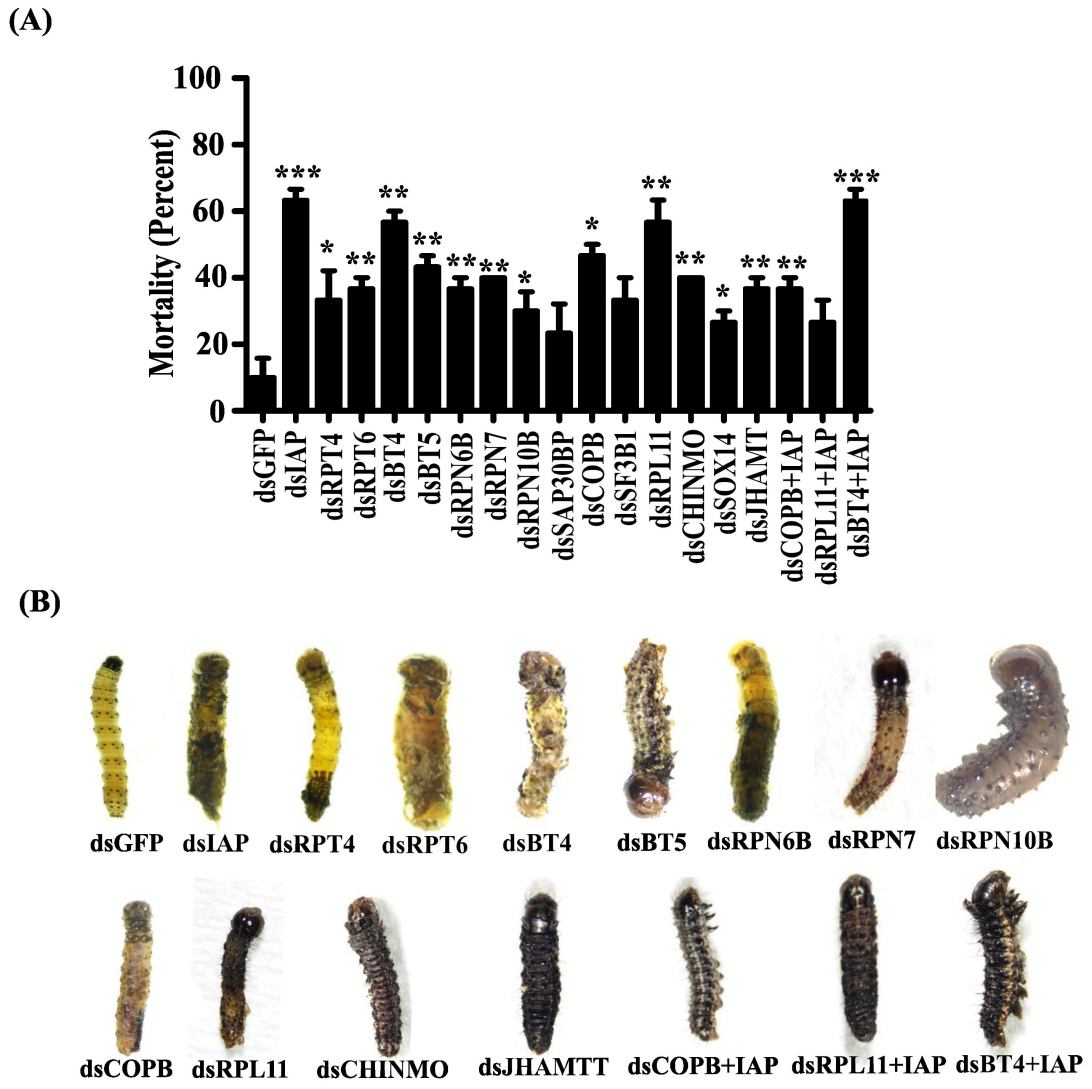
**(A)** Larval mortality caused by nanoformulated double-stranded RNA. Mortality was assessed following oral delivery of nanoformulated double-stranded RNA (dsRNA) targeting 14 candidate genes, with dsGFP as a negative control and dsIAP as a positive control, across three independent bioassays. Data are presented as the mean percent mortality ± standard error of the mean (SEM) across all bioassays. Each bioassay included 10 larvae, for a total of 30 per treatment. dsGFP showed minimal non-specific toxicity, whereas dsIAP induced consistently high mortality. Among the target genes tested, targeting proteasome subunit BT4 and ribosomal protein dsRPL11 resulted in significantly higher mortality (~50–70%). **(B)** Larval mortality and phenotypes in *S. frugiperda* following feeding of dsRNA nanoformulations targeting different genes. Larvae were fed on plants treated with nanoformulated dsRNA targeting selected candidate genes, and phenotype changes were photographed. Data are presented as mean ± SE. *P < 0.05; **P < 0.01; ***P < 0.001; ****P < 0.0001.

We expanded the analysis to identify additional candidate genes with the potential to improve or complement IAP-mediated mortality. Fourteen genes were selected based on the previous reports that their knockdown caused high mortality across multiple insect species (**Supplementary Table 2**). Fragments of genes representing diverse cellular functions and include regulatory subunit 10B (RPN10B), regulatory subunit 6B (RPN6B), Proteasome subunit beta type 4 (BT4), Proteasome subunit beta type 5 (BT5), regulatory subunit 7C (RPN7), non-ATPase regulatory subunits 6 and 4 (RPT6 and 4), SAP30-binding protein (SAP30BP), coatomer subunit beta (COPB), splicing factor 3B subunit 1 (SF3B1), 60S ribosomal protein L11 (RPL11), chinmo, juvenile hormone acid methyltransferase (JAHMT), and sox14 were amplified (**Supplementary Figure 1**) and dsRNAs prepared. Each dsRNA was evaluated in *S. frugiperda* larvae using nanoformulated dsRNA under optimized oral delivery conditions. To ensure reproducibility, bioassays were conducted in three independent experiments, each using 10 *S. frugiperda* neonates and 5 corn seedlings. The dsIAP served as a positive control. Larval mortality consistently plateaued at approximately 60% and 70% in dsIAP treatments in all three bioassays (**Figure 5**). Among the 14 candidate genes screened, BT4 and RPL11 emerged as the most potent targets, along with IAP. In the first bioassay, oral delivery of nanoformulated dsBT4 and dsRPL11 resulted in 60% and 70% larval mortality, respectively. While other dsRNAs, such as dsRPT4 and dsBT5, showed promise in one bioassay (50% mortality), their performance was inconsistent in the other bioassay, where dsRPT4 mortality dropped to 30%. In contrast, dsBT4 and dsRPL11 demonstrated superior reproducibility, maintaining 50% mortality in the second and third bioassays. The remaining candidate genes exhibited moderate mortality rates of 30%-50% across both bioassays.

Based on their consistent performance, dsBT4 and dsRPL11 were selected for dual gene-target formulation to determine whether co-silencing could enhance lethality. The dsBT4+dsIAP combination resulted in a significant increase in mortality, from 50% (single-target) to 60–70% in the dual-target assays. In contrast, the dsRPL11 + dsIAP combination did not show a similar increase, resulting in lower overall mortality than the dsBT4 + dsIAP combination. These results identify BT4 and RPL11 as critical genes in *S. frugiperda*, with dsBT4 showing potential for synergistic crop protection when combined with dsIAP. Targeting several additional genes, including BT5 and COPB, produced moderate but consistent mortality with mortality rates ranging from 45% to 50% (**Figure 5**). Larvae treated with dsGFP control formulations showed negligible mortality (~10%), confirming that the observed effects were sequence-specific and not attributable to nanoparticle or formulation toxicity.

### Morphological deformities in dsRNA-exposed *S. frugiperda*

Oral delivery of nano-formulated dsRNA induced distinct phenotypes that preceded or accompanied larval mortality. While control larvae (dsGFP) exhibited synchronized growth and successfully transitioned to the adult stage, treated groups showed significant developmental disruptions beginning on Day 6. In both experimental sets, BT4-targeted larvae exhibited persistent stunted growth, remaining significantly smaller than age-matched controls. In contrast, larvae treated with RPL11 and COPB exhibited severe melanization and visible loss of structural integrity, likely due to degradation of internal tissues and collapse of cellular transport mechanisms. These individuals often appeared sluggish and failed to maintain posture, indicating systemic physiological failure. Beyond these acute symptoms, a range of phenotypic abnormalities, including molting complications and body wall softening, were observed across the remaining candidate gene treatments. Mortality was recorded daily from Day 6 until pupation; notably, lethal effects were so pervasive that only larvae in treatments with negligible RNAi activity successfully reached the adult stage (**Figure 5**). The correlation between gene-specific silencing and morphological collapse underscores the high efficacy of the PLL/EGCG delivery system in disrupting essential larval development.

Overall, the comparative analysis showed that IAP-targeted formulations, particularly when combined with dsBT4, produced the most potent and consistent biological effects across all candidate genes evaluated. The superior performance of this dual-target strategy underscores the critical intersection of efficient nanoparticle-mediated delivery and strategic gene selection. Collectively, these results establish the PLL/EGCG/dsIAP and IAP+dsBT4 complex as a highly effective RNAi-based framework for managing *S. frugiperda*, providing a robust foundation for developing next-generation biopesticides.

## Discussion

The successful formation of PLL/EGCG/dsRNA nanoparticles observed in our studies is similar to that previously reported for catechin-based, polycationic nanocarriers for dsRNA delivery (8). The PLL/EGCG nanoparticles form stable, positively charged complexes that protect dsRNA from nuclease degradation and promote cellular uptake in *S. frugiperda* Sf9 cells (8). Our results extend these findings from *in vitro* systems to whole-organism oral delivery. The absence of free dsRNA in agarose gels suggests strong electrostatic interactions between dsRNA and the nanoparticle matrix, which likely shield dsRNA from alkaline gut conditions and dsRNase activity. High nuclease activity in the midgut lumen has been identified as a major barrier to RNAi in lepidopterans, including *S. frugiperda* (16–18). The protective effect of PLL/EGCG/dsRNA observed in the present study is consistent with previous reports employing alternative nanoparticle delivery systems for insect RNAi. For example, chitosan nanoparticles have been shown to enhance dsRNA stability and RNAi efficiency in the migratory locust, *Locusta migratoria* (19) by protecting dsRNA from degradation and facilitating cellular uptake. Similarly, lipid nanoparticle-based formulations have recently been reported by (20) to improve the stability and delivery of dsRNA in the RNAi-recalcitrant fall armyworm, *Spodoptera frugiperda*, thereby enhancing gene silencing activity. Consistent with these studies, our ex vivo midgut stability assay demonstrated that naked dsRNA was rapidly degraded in the lumen extract, whereas PLL/EGCG/dsRNA remained intact and recoverable following SDS-mediated dissociation. These findings suggest that protection against digestive degradation is a key mechanism by which nanoparticle carriers improve the performance of oral RNAi in lepidopteran insects. The ability of PLL/EGCG nanoparticles to maintain dsRNA integrity within the hostile midgut environment may increase the amount of biologically active dsRNA available for uptake, thereby contributing to the enhanced RNAi efficacy observed in this study. Similar protective effects have been reported for other nanoparticle systems, including chitosan, guanylated polymers, and star polycations, all of which improved dsRNA stability and uptake in lepidopteran midgut (21, 22).

Oral dsRNA delivery has long been recognized as a promising strategy for species-specific insect pest control; however, its application in lepidopteran insects has been consistently hampered by poor dsRNA stability, inefficient cellular uptake, and rapid degradation by gut nucleases (4, 23, 24). To address these barriers, nanoparticle-based delivery systems were developed as an effective strategy to enhance dsRNA stability, cellular uptake, and RNAi efficiency. Our previous work demonstrated that protamine–lipid–dsRNA nanoparticles markedly increased gene knockdown efficiency and induced high levels of larval mortality in *S. frugiperda* with the IAP as a target gene using the sugar droplet feeding assay (13), providing direct evidence that nanoparticle-mediated oral delivery can overcome key physiological limitations in this species.

The inhibitor of apoptosis (IAP) gene was selected as a benchmark RNAi target due to its essential role in regulating programmed cell death. Previous studies have consistently shown that IAP gene silencing induces apoptosis and mortality across multiple insect species (8, 25–27). In *S. frugiperda*, nanoparticle-mediated IAP knockdown has been reported to cause moderate to high larval mortality depending on the delivery system and exposure regime (8, 13). In the present study, optimized oral delivery of PLL/EGCG/dsIAP resulted in reproducible mortality, with a plateau at approximately 63%, and an 80% reduction in IAP mRNA levels. This level of knockdown exceeds that reported for naked dsRNA and is comparable to or higher than the mortality achieved with alternative nanoparticle platforms in *S. frugiperda* (protamine-lipid or star-polycation systems) (13, 22). Compared with previously reported nanocarrier systems such as protamine lipid or star polycation platforms, the PLL/EGCG nanoparticle system offers several potential advantages. Catechin-based poly-L-lysine nanoparticles have been shown to efficiently condense and protect dsRNA, while promoting cellular uptake through electrostatic interactions with insect gut epithelium. The inclusion of EGCG, a plant-derived polyphenol, may further enhance dsRNA stability and biocompatibility (28). Together, these features are likely to contribute to the enhanced RNAi efficacy observed in this study. However, direct comparative evaluations among different nanocarrier systems remain an important direction for future research. Knockdown efficiency varied among individual larvae, possibly reflecting differences in nanoparticle uptake and feeding behavior, resulting in heterogeneous dsRNA exposure. The discrepancy between high knockdown and incomplete mortality could be due to functional redundancy in apoptotic pathways or individual variability in dsRNA uptake, a phenomenon frequently observed in lepidopteran RNAi studies (4, 24).

In the whole seedlings bioassay, by the end of day 6 after adding larvae, control plants were almost completely defoliated. In contrast, nanoformulated dsIAP-treated seedlings showed only minor to moderate feeding damage, with the extent varying among the dsRNAs tested, consistent with differences in larval mortality (**Supplementary Figure 2**). However, after 6 days of treatment, most seedlings began to turn pale yellow and were not in good physiological condition. Therefore, the larvae were collected and transferred to a regular diet. These results confirm that the RNAi effect was triggered rapidly enough to arrest larval feeding and prevent economically significant crop damage. Overall, the present study demonstrates that PLL/EGCG-based nanoparticle formulations significantly enhance dsRNA delivery and RNAi efficacy in *S. frugiperda*, a species widely regarded as refractory to oral RNAi.

Further assessment of RNAi efficacy across multiple target genes, the proteasome subunit BT4 and the ribosomal protein RPL11 emerged as the most effective targets on par with IAP. Silencing of proteasome subunit BT4 and RPL11 resulted in approximately 56 percent mortality across all bioassays, comparable to that of the IAP-positive control. In contrast, RPL11 exhibited higher peak mortality in experiment 1 (~70%) but variability across experimental replicates. The robust and reproducible mortality observed following BT4 silencing is consistent with previous RNAi studies targeting proteasome components in other insect species. For example, RNAi-mediated knockdown of a 26S proteasome subunit in the Japanese beetle (*Popillia japonica*) increased mortality (28), demonstrating that proteasome disruption produces strong phenotypic effects on insect survival. Similarly, silencing of a 20S proteasome β subunit in the migratory locust (*Locusta migratoria*) impaired midgut homeostasis and reduced survival, underscoring the essential role of proteasome activity in maintaining cellular and tissue-level integrity (29). In addition, large-scale RNAi screening efforts in *Tribolium castaneum* have identified the proteasome pathway as one of the most lethal target classes, further supporting the proteasome as a conserved and highly vulnerable RNAi target across insect orders (30). Proteasome subunits have gained increasing attention as RNAi targets due to their function in protein turnover and cellular homeostasis. Our findings for BT4 align with recent literature, which shows that RNAi targeting proteasome subunits induced high mortality across diverse insect orders, including Coleoptera and Hemiptera (31, 32). In this context, the consistent performance of BT4 across independent assays in *S. frugiperda* extends these findings to a lepidopteran species demonstrating limited susceptibility to RNAi. RPL11 also achieved high mortality in the present findings; however, its variable performance contrasts with the reproducibility observed for BT4, reinforcing the relative robustness of proteasome-associated targets compared with ribosomal components. It indicates that basic intracellular components, such as ribosomal proteins, are primarily required for cellular homeostasis and have been proposed as attractive targets for RNAi-mediated pest control. In *T. castaneum*, the 60S ribosomal protein L11 is among the highly expressed core genes across life stages, highlighting its essential biological role (33). Furthermore, synergistic delivery of BT4 and IAP dsRNAs increased mortality, suggesting that simultaneous disruption of proteostasis and apoptosis may partially overcome compensatory mechanisms in *S. frugiperda*. Combining dsBT4 with dsIAP did not exceed the mortality ceiling observed for either target alone but consistently achieved the highest mortality in this study (~63%). Similar additive or plateauing effects have been reported in dual-gene RNAi studies, where targeting multiple essential pathways improves consistency but does not necessarily result in complete lethality (34, 35). These findings suggest that physiological or delivery-related constraints may impose an upper limit on the mortality achievable with oral RNAi in *S. frugiperda*. Although dual-target formulations did not exceed the observed mortality ceiling, combining targets improved consistency and robustness, suggesting that physiological limitations, rather than target potency, may constrain the maximal oral RNAi efficacy in this species. Together, these findings emphasize that successful RNAi-based pest control in lepidopterans depends on both optimized delivery systems and rational target selection across essential pathways.

Several additional genes, including the proteasome subunit BT5 and the coatomer subunit beta (COPB), caused moderate mortality, consistently ranging from 40% to 50% across the sets. While ribosomal and vesicle trafficking genes are known to be essential for insect development, their RNAi sensitivity is often highly species-specific and can be influenced by the efficiency of the systemic RNAi response (4, 36). In contrast, targets such as SF3B1 and SAP30BP showed minimal mortality, with SAP30BP at 23%. Despite previous reports of these genes’ effectiveness in other insect orders, our results underscore the importance of species-specific validation. The low efficacy of these targets in *S. frugiperda* suggests that either these specific mRNA transcripts are more stable or that the biological pathways they govern are more redundant in Lepidoptera, rendering them unsuitable as RNAi targets. The negligible mortality observed in dsGFP controls confirms that the PLL/EGCG nanoparticle system is not inherently toxic and that the observed effects are sequence specific, a critical requirement for regulatory approval of RNAi-based pest control products. While sustained dsRNA release was not directly quantified in this study, the structural design of the PLL/EGCG nanoparticles is expected to reduce rapid degradation and may facilitate prolonged availability of dsRNA following oral ingestion. Direct *in vivo* measurements of release kinetics will be the focus of future investigations. Collectively, our findings reinforce the concept that successful RNAi in lepidopterans requires both efficient delivery systems and effective target genes.

## Conclusions

In conclusion, these studies validate IAP as an RNAi target in *S. frugiperda* and identify BT4 as a new, effective target gene. PLL/EGCG-mediated dsRNA delivery shows nanoparticle-enabled RNAi as a viable method for fall armyworm control. This approach overcomes oral RNAi barriers in *S. frugiperda* by encapsulating dsRNA in nanoparticles, facilitating uptake, and activating RNA interference, resulting in gene knockdown and mortality. Screening revealed high mortality by targeting different cellular pathways, including the proteasome β-subunit BT4 and ribosomal protein RPL11, indicating multiple cellular processes as targets for RNAi. This work establishes a nanoparticle-delivery framework and identifies BT4, RPL11, and IAP as promising RNAi target genes. While tested in the lab, these results lay the groundwork for greenhouse and field evaluations of formulation stability and delivery efficiency, advancing RNAi-based fall armyworm management.

## Data Availability

The original contributions presented in the study are included in the article/**Supplementary Material**. Further inquiries can be directed to the corresponding author.

## References

[1] Sparks TC Nauen R . IRAC: mode of action classification and insecticide resistance management. Pestic Biochem Physiol. (2015) 121:122–8. doi: 10.1016/j.pestbp.2014.11.014 26047120

[2] Tudi M Daniel Ruan H Wang L Lyu J Sadler R Connell D . Agriculture development, pesticide application and its impact on the environment. Int J Environ Res Public Health. (2021) 18:1112. doi: 10.3390/ijerph18031112 33513796 PMC7908628

[3] He L Huang Y Tang X . RNAi-based pest control: production, application and the fate of dsRNA. Front Bioeng Biotechnol. (2022) 10:1080576. doi: 10.3389/fbioe.2022.1080576 36524052 PMC9744970

[4] Zhu KY Palli SR . Mechanisms, applications, and challenges of insect RNA interference. Annu Rev Entomol. (2020) 65:293–311. doi: 10.1146/annurev-ento-011019-025224 31610134 PMC9939233

[5] Palli SR . RNAi turns 25: contributions and challenges in insect science. Front Insect Sci. (2023) 3:1209478. doi: 10.3389/finsc.2023.1209478 38469536 PMC10926446

[6] Shukla JN Kalsi M Sethi A Narva KE Fishilevich E Singh S . Reduced stability and intracellular transport of dsRNA contribute to poor RNAi response in lepidopteran insects. RNA Biol. (2016) 13:656–69. doi: 10.1080/15476286.2016.1191728 27245473 PMC4962799

[7] Kebede M Fite T . RNA interference (RNAi) applications to the management of fall armyworm, Spodoptera frugiperda (Lepidoptera: Noctuidae): its current trends and future prospects. Front Mol Biosci. (2022) 9:944774. doi: 10.3389/fmolb.2022.944774 36158573 PMC9490220

[8] Dhandapani RK Gurusamy D Palli SR . Development of catechin, poly-l-lysine, and double-stranded RNA nanoparticles. ACS Appl Bio Mater. (2021) 4:4310–8. doi: 10.1021/acsabm.1c00109 35006843

[9] Palli SR Koo J Moola AK . RNA interference: basic science to applications in pest management. In: Comprehensive Molecular Insect Science Amsterdam: Elsevier (2025). p. V5:160–79. doi: 10.1016/B978-0-323-95424-2.00063-7

[10] Ahmadzada T Reid G McKenzie DR . Fundamentals of siRNA and miRNA therapeutics and a review of targeted nanoparticle delivery systems in breast cancer. Biophys Rev. (2018) 10:69–86. doi: 10.1007/s12551-017-0392-1 29327101 PMC5803180

[11] Mahmoodi Chalbatani G Dana H Gharagouzloo E Grijalvo S Eritja R Logsdon CD . Small interfering RNAs (siRNAs) in cancer therapy: a nano-based approach. Int J Nanomed. (2019) 14:3111–28. doi: 10.2147/IJN.S200253 PMC6504672 31118626

[12] Wu X Li W Xu Q Yi X Zhang D He M . Delivery of dsRNA to improve RNAi efficiency by cell-penetrating disulfide polymer for Spodoptera frugiperda control. (2024) 166:104881. doi: 10.21203/rs.3.rs-5052291/v1 40945706

[13] Dhandapani RK Gurusamy D Palli SR . Protamine–lipid–dsRNA nanoparticles improve RNAi efficiency in the fall armyworm, Spodoptera frugiperda. J Agric Food Chem. (2022) 70:6634–43. doi: 10.1021/acs.jafc.2c00901 35612305

[14] Buer B Dönitz J Milner M Mehlhorn S Hinners C Siemanowski‐Hrach J . Superior target genes and pathways for RNAi‐mediated pest control revealed by genome‐wide analysis in the beetle Tribolium castaneum. Pest Manage Sci. (2025) 81:1026–36. doi: 10.1002/ps.8505 PMC11716340 39498580

[15] Hwang HS Kang HC Bae YH . Bioreducible polymers as a determining factor for polyplex decomplexation rate and transfection. Biomacromolecules. (2013) 14:548–56. doi: 10.1021/bm301794d 23259985 PMC3570707

[16] Vélez AM Fishilevich E . The mysteries of insect RNAi: a focus on dsRNA uptake and transport. Pestic Biochem Physiol. (2018) 151:25–31. doi: 10.1016/j.pestbp.2018.08.005 30704709

[17] Cooper AM Silver K Zhang J Park Y Zhu KY . Molecular mechanisms influencing efficiency of RNA interference in insects. Pest Manage Sci. (2019) 75:18–28. doi: 10.1002/ps.5126 29931761

[18] Koo J Palli SR . dsRNase1 contribution to dsRNA degradation activity in the Sf9 cells conditioned medium. Front Insect Sci. (2023) 3:1118775. doi: 10.3389/finsc.2023.1118775 38469530 PMC10926405

[19] Liu Y Zhang J Li S Chai L Chang BH Malak M . Chitosan nanoparticle-mediated delivery of dsRNA for enhancing RNAi efficiency in Locusta migratoria. Pest Manage Sci. (2025) 81:5260–9. doi: 10.1002/ps.8423 40342233

[20] Kaarow M Graser L Knorr E Windfelder A Geisler P Steiniger F . Enhancing the delivery and stability of lipid nanoparticle–dsRNA formulations in the RNAi-recalcitrant fall armyworm (Spodoptera frugiperda). Front Insect Sci. (2026) 6. doi: 10.3389/finsc.2026.1770055 PMC13062331 41970227

[21] Christiaens O Tardajos MG Martinez Reyna ZL Dash M Dubruel P Smagghe G . Increased RNAi efficacy in Spodoptera exigua via the formulation of dsRNA with guanylated polymers. Front Physiol. (2018) 9:316. doi: 10.3389/fphys.2018.00316 29670535 PMC5894468

[22] Chao Z Ma Z Zhang Y Yan S Shen J . Establishment of star polycation-based RNA interference system in all developmental stages of fall armyworm Spodoptera frugiperda. Entomol Gen. (2023) 43:127. doi: 10.1127/entomologia/2023/1906

[23] Wang K Peng Y Pu J Fu W Wang J Han Z . Variation in RNAi efficacy among insect species is attributable to dsRNA degradation *in vivo*. Insect Biochem Mol Biol. (2016) 77:1–9. doi: 10.1016/j.ibmb.2016.07.007 27449967

[24] Huvenne H Smagghe G . Mechanisms of dsRNA uptake in insects and potential of RNAi for pest control: a review. J Insect Physiol. (2010) 56:227–35. doi: 10.1016/j.jinsphys.2009.10.004 19837076

[25] Zhang J Khan SA Heckel DG Bock R . Next-generation insect-resistant plants: RNAi-mediated crop protection. Trends Biotechnol. (2017) 35:871–82. doi: 10.1016/j.tibtech.2017.04.009 28822479

[26] Maximo WPF Howell JL Mogilicherla K Basij M Chereddy S Palli SR . Inhibitor of apoptosis is an effective target gene for RNAi-mediated control of Colorado potato beetle, Leptinotarsa decemlineata. Arch Insect Biochem Physiol. (2020) 104:e21685. doi: 10.1002/arch.21685 32350927

[27] Mogilicherla K Howell JL Palli SR . Improving RNAi in the Brown Marmorated Stink Bug: identification of target genes and reference genes for RT-qPCR. Sci Rep. (2018) 8:3720. doi: 10.1038/s41598-018-22035-z 29487333 PMC5829169

[28] Lucetti G Abbà S Pesavento G Fanelli E De Luca F Battagini E . RNAi‐mediated gene silencing of a 26S proteasome subunit increases mortality of the Japanese beetle Popillia japonica. Pest Manage Sci. (2026) 82:1066–78. doi: 10.1002/ps.8564 PMC12713707 41048186

[29] Yang Y Wang M Guo W Zhu Q Zhang D Zhang X . RNAi of 20S proteasome β subunit 3 inhibits midgut homeostasis and the survival of the migratory locust, Locusta migratoria. Pest Manage Sci. (2025) 81:8533–42. doi: 10.1002/ps.70157 40852919

[30] Ulrich J Dao VA Majumdar U Schmitt-Engel C Schwirz J Schultheis D . Large scale RNAi screen in Tribolium reveals novel target genes for pest control and the proteasome as prime target. BMC Genomics. (2015) 16:674. doi: 10.1186/s12864-015-1880-y 26334912 PMC4559001

[31] Canuto F Taliano E Marzachì C Bosco D Galetto L . RNAi-mediated silencing of a regulatory subunit of the 26S proteasome induces mortality and female sterility in leafhopper vectors of grapevine Flavescence dorée phytoplasmas. J Pest Sci. (2025) 98:2625–34. doi: 10.1007/s10340-025-01961-8 30311153

[32] Rodrigues TB Mishra SK Sridharan K Barnes ER Alyokhin A Tuttle R . First sprayable double-stranded RNA-based biopesticide product targets proteasome subunit beta type-5 in Colorado potato beetle (Leptinotarsa decemlineata). Front Plant Sci. (2021) 12:728652. doi: 10.3389/fpls.2021.728652 34887882 PMC8650841

[33] Perkin LC Oppert B . Gene expression in Tribolium castaneum life stages: identifying a species-specific target for pest control applications. PeerJ. (2019) 7:e6946. doi: 10.7717/peerj.6946 31198628 PMC6535216

[34] Zotti M Smagghe G . RNAi technology for insect management and protection of beneficial insects from diseases: lessons, challenges and risk assessments. Neotrop Entomol. (2015) 44:197–214. doi: 10.1007/s13744-015-0291-8 26013264

[35] Yan S Ren BY Shen J . Nanoparticle‐mediated double‐stranded RNA delivery system: a promising approach for sustainable pest management. Insect Sci. (2021) 28:21–34. doi: 10.1111/1744-7917.12822 32478473

[36] Whyard S Singh AD Wong S . Ingested double-stranded RNAs can act as species-specific insecticides. Insect Biochem Mol Biol. (2009) 39:824–32. doi: 10.1016/j.ibmb.2009.09.007 19815067

