# Supplementary material for: Improving RNAi in the fall armyworm, Spodoptera frugiperda
Source: Front Insect Sci. 2026 Jun 30;6:1820222. doi: 10.3389/finsc.2026.1820222 (PMC13364971; doi:10.3389/finsc.2026.1820222)
Supplement: Supplementary file 1 [file SupplementaryFile1.pdf]

Supplementary Information

Improving RNAi in the Fall Armyworm, *Spodoptera frugiperda*

Anil Kumar Moola & Subba Reddy Palli*

Department of Entomology, Martin Gatton College of Agriculture, Food and Environment, University of Kentucky, Lexington, KY, 40546, USA

*** Corresponding author. E-mail address: rpalli@uky.edu (S.R. Palli).**

Table 1S. Size, PDI, and Charge of PLL/EGCG/dsRNA nano formulations.

Size	PDI	Charge
311.63 ± 64.08	0.35 ± 0.10	27.12 ± 0.61

Table 2S Primers used in the present study

**Gene Name (*S. frugiperda*)**	**Accession Number**	**Size (bp)**	**Primer Sequence (5' to 3')**
26S proteasome non-ATPase regulatory subunit 4 F (RPT4 F)	XM_035602482.2	386	GTATTTGTCGGTTCGCCAGT
26S proteasome non-ATPase regulatory subunit 4 R (RPT4 R)			GACTCTTCCTCTTGGCGTTG
26S proteasome non-ATPase regulatory subunit 6 non-ATPase regulatory subunit 6 F (RPT6 F)	XM_035590595.2	306	CAAGGCTGCAGATTTGTTCA
26S proteasome non-ATPase regulatory subunit 6 non-ATPase regulatory subunit 6 R (RPT6 R)			ACGTAGTGCTGGTAGTGGGG
Proteosome subunit Beta Type 4 F (BT4 F)	XM_035599694.2	409	CAGGCGCCTTCTACAACTTC
Proteosome subunit Beta Type 4 R (BT4 R)			TAGTTGCTCCACAAGGGGTC
Proteosome subunit Beta Type 5 F (BT5 F)	XM_035596328.2	343	CCCGTAGACGCACTAGCTTC
Proteosome subunit Beta Type 5 R (BT5 R)			CACGGTCCCAGTACACACAG
26S proteasome regulatory subunit 6B F (RPN6BF)	XM_035602694.2	375	GCCAGTTCCTTGAAGCAGTC
26S proteasome regulatory subunit 6B R (RPN6BR)			AGTTTTGCCACATCCAGGAG
26S proteasome regulatory subunit 7 F (RPN7 F)	XM_035587768.2	321	CACAAAAATCATCAATGCGG
26S proteasome regulatory subunit 7 R (RPN7 R)			CCCAGCTTCACGAACTTCTC
26S proteasome regulatory subunit 10B F (RPN10B F)	XM_035586443.2	302	ACACTCACCATCATGAGGCA
26S proteasome regulatory subunit 10B R (RPN10B R)			ACGATGGCTGAGGATACCAC
SAP30-binding protein F (SAP30BP F)	XM_035579197.2	250	TGTGAAGACGGTGTGACCAT
SAP30-binding protein R (SAP30BP F)			ATGATTCTTTGCCCCACTTG
Coatomer subunit beta F (COPB F)	XM_035575056.2	351	CCTGCGCTTCCTCTGTAAAC
Coatomer subunit beta R (COPB R)			GCGTGGCAAACCTTGTAAAT
Splicing factor 3B subunit 1 F (SF3B1 F)	XM_050703182.1		AACAGTGGTTGGGCTGAAAC

Splicing factor 3B subunit 1 R (SF3B1 R)		372	ATCAGTGTAGGGACGGTTGC
60S ribosomal protein L11 F (RPL11 F)	XM_035588345.2	308	CCAACAGCCAGTGTTCTCAA
60S ribosomal protein L11 R (RPL11 R)			TCTTGCGTCTTCTGTGTGCT
Sox 14 F (Sox 14 F)	XM_035590597.2	330	TCTTGGTGCACAGTGGAAAG
Sox 14 R (Sox 14 R)			CGTAGCGAGGGTAGCCATAG
Chinmo F	XM_035589772.2	493	TTATTTGACTCAGCACCCCC
Chinmo R			GCCTGTAATTCCTTGCGTGT
Juvenile hormone acid O-methyltransferase F (JAHMT F)	XM_035578501.2	304	CGTGGCTAATTCACTGCAAA
Juvenile hormone acid O-methyltransferase R (JAHMT R)			AGAACACGTGATGGAACGCT

*T7 promoter (TAATACGACTCACTATAGGG) sequence added on the 5’ end of primer sequence

Figure 1S

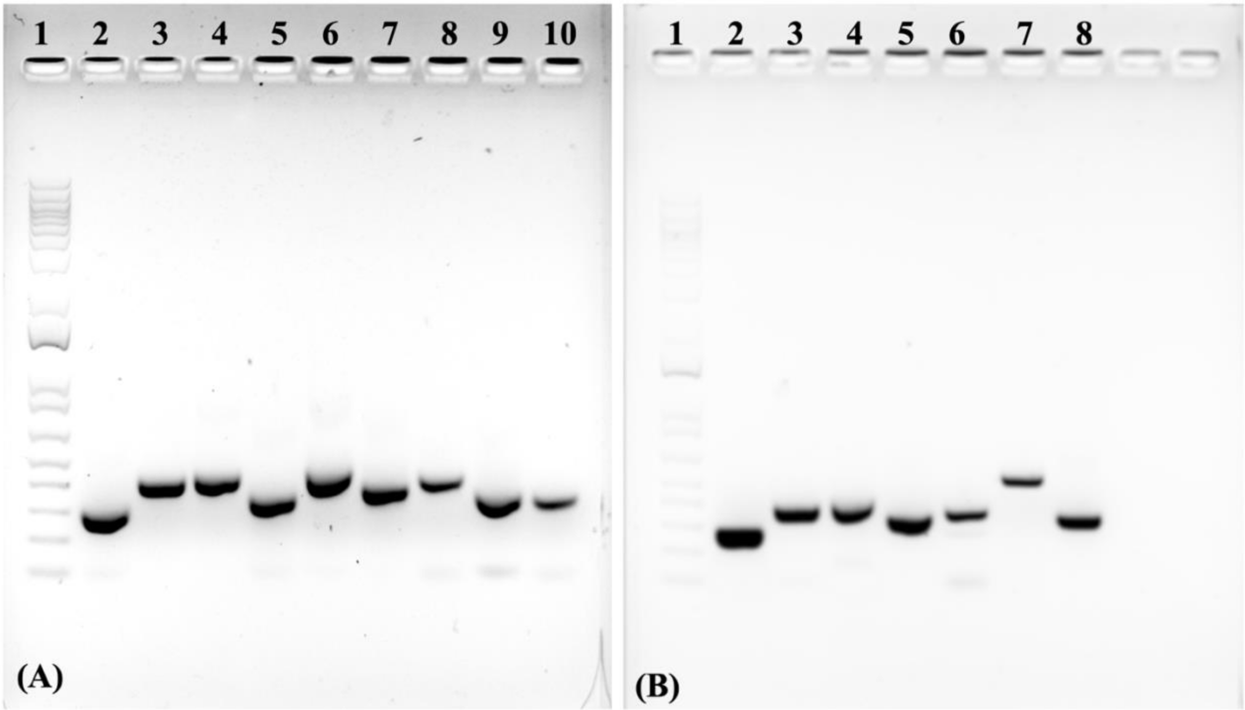

Figure 1S. Verification of amplicon size by agarose gel electrophoresis.

Amplicon size of 16 target genes was confirmed by agarose gel electrophoresis, with each PCR reaction yielding a single band of the expected size.
(A) Lane 1: 1 kb DNA ladder; Lanes 2–10: GFP, IAP, RPT4, RPT6, BT4, BT5, RPN 6B, RPN7, and RPN10B.
(B) Lane 1: 1 kb DNA ladder; Lanes 2–8: SAP30BP, COPB, SF3B1, RPL11, SOX14, Chinmo, and JAHMT.

Figure 2S

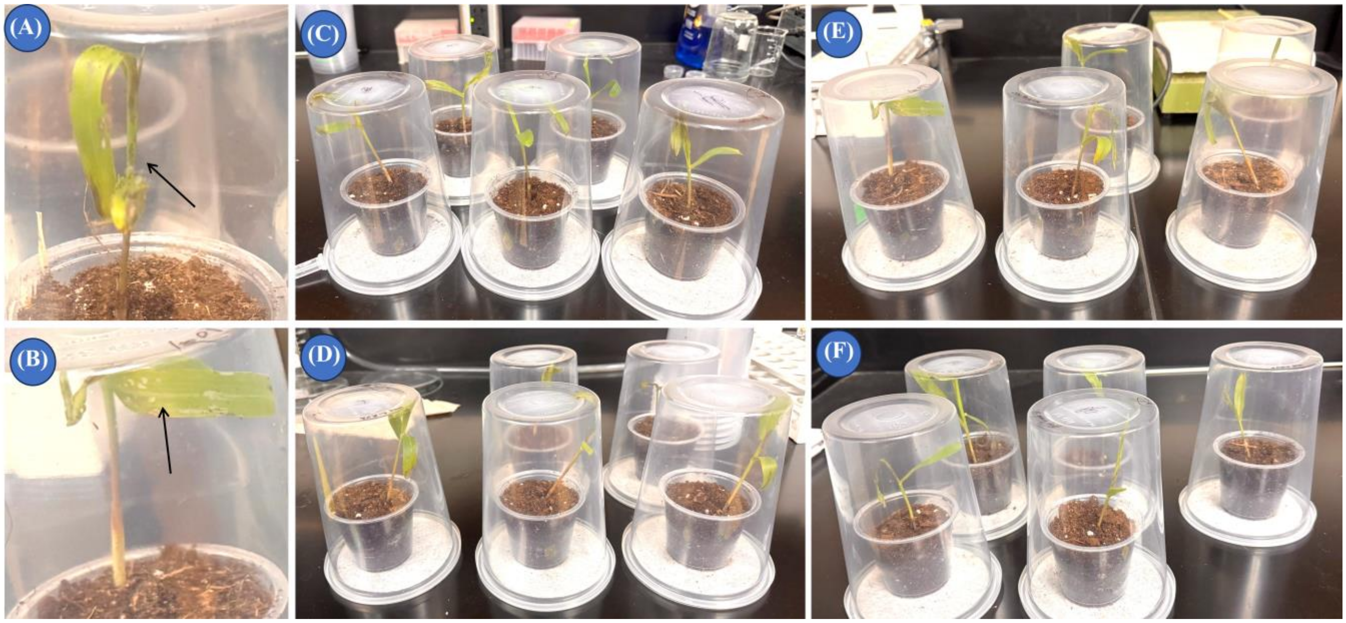

Figure 2S. Feeding damage on whole seedlings following dsRNA treatments.
(A) Representative images of a dsGFP-treated seedling (control) on day 4 post-application, showing substantial defoliation and leaf loss. (B) Representative image of a nanoformulated dsIAP-treated seedling on day 4, exhibiting only minor feeding damage. Overall, control plants were more severely defoliated compared to dsIAP-treated seedlings. (C–F) Representative images of seedlings treated with nano-formulated BT4, IAP, COPB, and RPL11 dsRNAs, respectively, showing variable levels of feeding damage.
